# Physician Burnout: Coaching a Way Out

**DOI:** 10.1007/s11606-014-3144-y

**Published:** 2014-12-20

**Authors:** Gail Gazelle, Jane M. Liebschutz, Helen Riess

**Affiliations:** 1 Division of General Internal Medicine and Primary Care, Brigham and Women’s Hospital, Harvard Medical School, POB 669, Brookline, MA 02446 USA; 2 Section of General Internal Medicine, Boston Medical Center, Boston University School of Medicine, Boston, MA USA; 3 Empathy and Relational Science Program, Department of Psychiatry, Massachusetts General Hospital, Harvard Medical School, Boston, MA USA

**Keywords:** physician burnout, physician coaching, professional coaching, physician wellness, physician resilience, executive coaching, health coaching, physician leadership coaching, work-life balance, resilience

## Abstract

Twenty-five to sixty percent of physicians report burnout across all specialties. Changes in the healthcare environment have created marked and growing external pressures. In addition, physicians are predisposed to burnout due to internal traits such as compulsiveness, guilt, and self-denial, and a medical culture that emphasizes perfectionism, denial of personal vulnerability, and delayed gratification. Professional coaching, long utilized in the business world, provides a results-oriented and stigma-free method to address burnout, primarily by increasing one’s internal locus of control. Coaching enhances self-awareness, drawing on individual strengths, questioning self-defeating thoughts and beliefs, examining new perspectives, and aligning personal values with professional duties. Coaching utilizes established techniques to increase one’s sense of accomplishment, purpose, and engagement, all critical in ameliorating burnout. Coaching presumes that the client already possesses strengths and skills to handle life’s challenges, but is not accessing them maximally. Although an evidence base is not yet established, the theoretical basis of coaching’s efficacy derives from the fields of positive psychology, mindfulness, and self-determination theory. Using a case example, this article demonstrates the potential of professional coaching to address physician burnout.

Changes in medical practice have augmented stressors for already overburdened physicians, including increased scrutiny, accountability, time constraints, and increasing role definition by non-physicians, all with a concomitant decrease in workplace control.^1^–^3^ The growing mismatch between workload and sense of control,^4^ along with unique physician personality factors, contributes to widespread burnout.^5^–^7^ One method to address burnout is professional coaching, drawing on strengths, questioning assumptions, and aligning values with purpose, to increase life and career satisfaction. This article examines the etiology of burnout, using a case example to demonstrate how coaching enhances one’s internal locus of control, critical in addressing burnout.

*Dr. Greenley,* 1 *54, is a well-respected internist. Recently, he has become short-tempered with staff, persistently late with paperwork, exhausted, and emotionally distant. Dwelling on negative aspects of his personality, he feels like an imposter. Greenley feels disconnected from his earlier sense of purpose. Piano and tennis, past enjoyable pursuits, are long lost.*

Burnout is characterized by a low sense of personal accomplishment, emotional exhaustion, cynicism and depersonalization.^4^,^8^,^9^ Maslach, a pioneering burnout researcher, noted that burnout starts when “energy turns into exhaustion, involvement turns into cynicism, and efficacy turns into ineffectiveness.”^9^ ^, p.186^ Numerous studies note rates of burnout between 25 % and 60 % in a wide spectrum of specialties.^5^,^10^–^14^ A 2012 U.S. study found that 47 % of 7,288 physicians experienced burnout, considerably higher than in the general population.^8^ Of note, over 50 % of 578 general internists experienced burnout, second only to emergency physicians.^8^

In addition to deleterious effects on physician well-being, burnout contributes to decreased physician retention,^15^ and correlates with self-reported suboptimal care, patient noncompliance, and medical errors.^7^,^16^,^17^ Moreover, physician distress contributes to staff turnover, failing morale, and decreased cohesiveness of the entire healthcare enterprise.^18^,^19^

The etiology of physician burnout not only includes growing external demands and decreased workplace control; socialization factors also play a role. Medical training emphasizes perfectionism, denial of personal vulnerability, and delayed gratification.^6^,^7^,^17^ Traits such as compulsiveness, guilt, and self-denial may facilitate success in medical education and training; however, in a long-term career, these same traits can fuel feelings of inadequacy. Set in a professional culture that stigmatizes weakness and self-care,^6^,^7^,^17^,^20^ these factors contribute to burnout, when external pressures overwhelm internal sense of control.^4^,^7^

*A friend of Dr. Greenley’s, a pharma executive, suggested coaching to improve his workplace function and state of mind.*

Professional coaching, long used by executives and making inroads within the medical profession,^21^–^28^ may lessen burnout. Coaching involves “partnering with clients in a thought-provoking and creative process that inspires them to maximize their personal and professional potential”.^29^ Coaching presupposes sufficient inner resources and the necessary expertise to tackle life challenges, and provides the guidance to harness these internal mechanisms.^30^–^32^ A core coaching construct is amplifying a client’s internal locus of control, defined as the belief that one’s actions have as much or more impact on life outcomes than external forces or individuals.^33^ Studies in a variety of professions note an inverse correlation between internal locus of control and burnout.^34^,^35^ In addition, coaching increases self-efficacy and self-determination, vital counterbalances to burnout,^4^,^36^ and critical for physicians rapidly losing workplace control. The overarching premise of professional coaching, in fact, is that people have more control over their life circumstances and satisfaction than they typically realize.

At the heart of coaching lies an iterative process of examining seemingly fixed thoughts and circumstances.^30^–^32^ Clients learn to question automatic thoughts, beliefs, and perceptions, thus discerning between facts, assumptions, and interpretations. Consciously shifting perspective is another technique for revealing new options for action, thus increasing choice and control. By exercising authority over their thoughts and beliefs, people can move from reactivity to purposeful response. Enhanced self-reflection and self-awareness are key to improving physician resilience,^7^,^20^,^37^–^40^ the flexibility to withstand and bounce-back from external stressors, and are foundational to the coaching enterprise.^31^,^41^

Coaching also helps expose and challenge negative emotional patterns, including self-defeating inner dialogue (e.g., “People think I’m a great physician, but they don’t see the real me.”) In addition, coaches encourage clients to focus awareness of their cognitive, emotional, and physical experience in the present, a central tenet of mindfulness.^42^–^45^

*Dr. Greenley contacted a certified coach specializing in physician clients. After completing life-balance* (Fig. 1) *and strengths assessments, he began 1-hour biweekly sessions and identified three goals: regaining sense of purpose, improving anger management, and enhancing work-life balance.* (Table 1).

**Figure 1.**
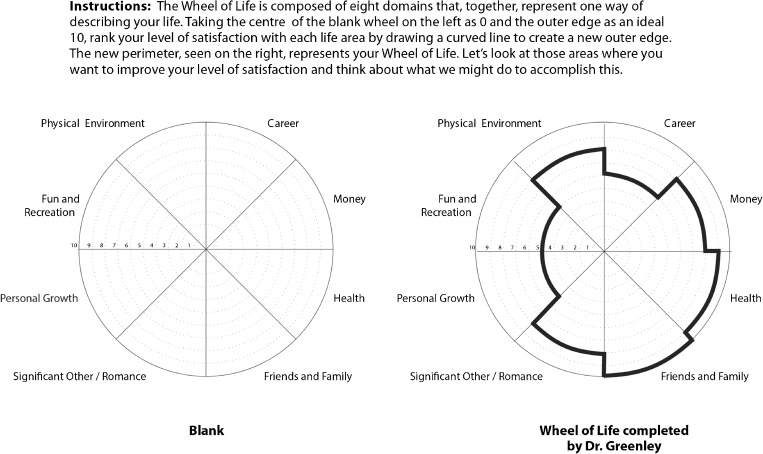
Wheel of life exercise.

**Table 1.** Case Study: Issues Addressed and Coaching Dialogue Examples

Dr. Greenley’s issues	Coach’s input	Dr. Greenley’s responses	Results
Fatigue, low sense of accomplishment: *“The demands on my time are too much; I’m exhausted and not accomplishing anything.”*	*“What’s another way of looking at this?”* *“What’s a new viewpoint?”* *“Will you start a ‘got-done’ list? Nightly, write down three things that went well.”*	*“I guess I helped my patients, that’s true. And, I felt good about what I said in the meeting.”* *“Maybe that I don’t need to be so hard on myself?”* *“I’ll try it, since I feel even more defeated thinking about what’s hanging over me.”*	1. Focus shifts from overwhelmed to appreciation of accomplishments. 2. Dr. Greenley’s sense of engagement with his work is fostered. 2. Realistic emphasis on the positive decreases stress and contributes to life balance.
Self-doubt: “*I should be smarter and more efficient. I don’t have what it takes.”*	*“I’m hearing a ‘not-good-enough’ message. How are you experiencing this right now?”* “*If we could wipe the slate clean, what would you do differently going forward?”*	*“It’s like my father’s voice inside my head. He was always saying that my older sister was the smart one.”* *“I’d start reminding myself that I’m just as smart as her. Look at me, I’m a well-respected internist.”*	1. Focusing on Dr. Greenley’s experience in the present leads to awareness of the source of the belief. 2. Orienting toward the future, the coach helps Dr. Greenley institute a more affirming internal message.
Compromised relationships: *“I get angry with staff and sometimes lose it. We’ve worked together for years; I don’t know what gets into me.”*	*“I hear disappoint-* *ment in yourself.”* *“What happens physically just before you lose control?”* *“When you experience this, is there another response?”*	*“Treating them this harshly is against my values.”* *“My heart races and it feels like my head will explode.”* *”I could put my hand on my heart and take a deep breath.”*	1. Connecting values with actions provides motivation for change. 2. Enhancing physical mindfulness reveals a “tipping point.” 3. Practice reinforces positive countermeasures.
Cynicism, decreased sense of purpose: *“I’m like a hamster on a wheel. Why go on?”*	*“What gives your work value and meaning?”* *“What energizes you?”*	*“My relationships with patients have always been what is meaningful for me.”* *“Playing piano brought me peace and energy, but I just don’t have any time.”*	1. Existing sources of professional meaning are highlighted. 2. Dr. Greenley finds a source of calm and revitalization. The coach encourages playing 15 minutes a day, helping Dr. Greenley glimpse control he *can* exert over his time, as well as his ability to effect change.
Inattention to personal health: *“I’d feel better if I played tennis regularly, but I never do.”*	*“Will you put tennis on your calendar twice this week?”*	*“That seems contrived, but yes.”*	1. Scheduling personal activities elevates their importance. 2. Dr. Greenley is held accountable for wellness behaviors.

Coaches help clients clarify values and align them with professional and personal goals, an objective of known importance in decreasing physician burnout.^7^,^16^,^19^ Rather than assign uninspiring to-do lists, coaches build motivation by eliciting solutions from clients, thus increasing personal investment, and making next steps obvious, possible, and even invigorating.^30^–^32^

Helping clients identify, bolster, and apply strengths in challenging situations is another technique, important in a medical culture known to malign personal weakness. Coaching applies techniques from the field of positive psychology, the scientific study of the strengths and virtues that enable optimal functioning of individuals and communities. Without minimizing painful emotions, positive psychology emphasizes engagement, meaning, and accomplishment.^46^,^47^ Engagement, “characterized by energy, involvement, and efficacy—the direct opposites of the three burnout dimensions,”^4^ is particularly important. While deepening engagement with work, coaches also attempt to revive creative pursuits (e.g., music, art, writing) and hobbies, which can serve as meaningful sources of renewal.^20^,^48^

Other successful interventions for physician burnout include many strategies embedded in professional coaching. Mindfulness training decreases burnout in physicians,^42^,^44^ as do discussion groups and other means of promoting self-reflection and awareness.^20^,^37^,^38^ Other aspects of coaching that appear promising for ameliorating physician burnout include aligning values with professional duties and increasing one s sense of purpose and engagement.^3^,^7^,^16^,^48^

*Dr. Greenley’s strengths assessment identified curiosity and gratitude as his foremost strengths. After receiving complaints about his anger, the coach and Dr. Greenley brainstormed ways to leverage his strengths with specific behaviors. For example, when feeling short-tempered with staff, he pushed himself to be more curious about their experience.*

Co-creating action steps and maintaining accountability are essential for successful coaching. Coaches hold clients to mutually agreed-upon actions, promoting active experimentation and self-discovery, teaching self-discipline, and helping to build on small successes to create rapid and sustainable change.

Integral for coaching and vitally important for quality patient care and the overall integrity of health systems is helping physicians reach their highest potential.^25^,^49^ From a nonjudgmental and championing stance, coaches help clients envision and move toward peak life and work performance.

It is important to compare coaching with other behavioral interventions. Cognitive-behavioral therapy (CBT,) a diagnosis-driven and goal-oriented method of improving coping, modifies thought patterns to improve coping and encourages clients to question whether negative thoughts are actually untested assumptions. Unlike coaching, CBT is classically delivered in a prescriptive and standardized manner.^50^ The professional coaching emphasis on wellness, enhanced function, accountability, and achieving goals differs from the pathology-based and diagnosis-based fields of traditional psychology and psychiatry, and thus carries less stigma. Coaching also considers the client the expert on their life, evoking wisdom from clients’ experience, as opposed to the teaching and advising characteristic of mentoring.^51^

Although all involve behavioral change, coaching is not psychological treatment and is oriented toward high-functioning individuals. Coaching is not appropriate with active psychiatric illness (such as major depression, psychosis, or obsessive compulsive disorder), and not effective in the setting of active substance abuse. Coaches are not trained mental health professionals and should have a low threshold for recommending psychological and/or psychiatric evaluation. Since burnout and depression often co-exist, in the absence of a major depressive episode, physicians can choose whichever approach they prefer, as overlap exists.

Coaching takes place in person or by phone. For physicians suffering from burnout, weekly or biweekly 1-hour sessions would likely be required for 6 to 12 months.

*The highly supportive coaching partnership promoted gradual change. Dr. Greenley adopted more effective responses to anger triggers. A heightened sense of purpose, agency, and accomplishment led to greater efficiency in paperwork. He began playing tennis and piano again. Most significantly, he now viewed his daily accomplishments as personal victories providing ballast against what once seemed like immutable oppression. Although his external circumstances remained unchanged, he became more energized and hopeful. After 9 months, he switched to monthly coaching sessions and continued his growth.*

Widely employed in the business world, studies reveal financial return of 2.2–5.7 times on investment.^52^,^53^ In addition, coaching can strengthen diverse professional skills, including decisiveness, time management, productivity, communication, leadership, and teamwork.^52^–^55^

## COACHING: EMERGING DATA

Coaching physicians is an emerging field; scant supporting evidence is currently available. The only studies with physician subjects involve peer-coaching, a model distinct from professional coaching.^56^,^57^ A pilot study of four nurses and physical therapists demonstrated improvement in job satisfaction and self- efficacy.^58^ Physician coaching case reports exist, both to improve communication skills^23^ and for physician leadership.^28^

Recent meta-analyses of coaching in non-healthcare corporate settings showed improvement in well-being, self-efficacy, and goal-directed self-regulation; however, heterogeneity of interventions and mixed study quality limits applicability.^55^,^59^

Research from the field of wellness coaching provides evidence that the professional coaching model can build self-efficacy, and effect behavioral and attitudinal change.^60^ For example, two studies involving 65 diabetic patients found greater medication adherence, greater sense of control over disease, and decreased A1C.^61^,^62^ A study of 24 adults with spinocerebellar degeneration demonstrated improved goal setting and self-efficacy scores.^63^ Professional coaching techniques were intentionally embedded in a randomized, controlled trial of resident empathy. Drawing from residents’ core values, and training in mindfulness and self-other-awareness techniques, this research demonstrated significant improvement in patient satisfaction.^64^

The authors identified only one study utilizing professional coaching with burnout as an outcome measure, finding that seven to nine coaching sessions decreased burnout and increased life satisfaction.^65^

Although more data is needed, influences from positive psychology, mindfulness, and self-determination and self-efficacy theories are embedded in the practice of coaching, and are receiving increasing attention in the medical literature.^19^,^44^,^66^–^69^ These contributions inform the early foundation of coaching’s potential in addressing physician burnout.

In particular, self-determination theory, seen as a guide for medical education,^70^,^71^ emphasizes the centrality of internal autonomy in maintaining motivation and satisfaction,^72^ postulating that full engagement stems from enhanced self-awareness, and alignment of internal aspirations with duties.^4^,^72^ Similarly, Maslach identifies internalized locus of control, engagement, value alignment, and positive psychology tenets to overcome burnout.^4^

While coaching has the potential to help physicians, limitations include financial cost, typically born by the physician. In addition, motivation, essential for any behavioral and attitudinal change, is required. Further, coaching cannot alter the many external factors contributing to burnout.^73^

Training standards for coaches are essential. Given physicians’ vital societal role, standards for physician coaching are needed, including accredited training, certification, ongoing supervision, periodic recertification, and continuing education. Training in psychiatry or psychology alone, or providing non-physician coaches superficial education about healthcare, will not suffice.

### Future Directions

The role of coaching in managing physician burnout warrants further exploration, due to its accessibility and potential for return on investment. Well-designed research studies will be critical going forward.

## CONCLUSION

The case of Dr. Greenley demonstrates how coaching can provide a means of increasing self-awareness, aligning personal values with professional duties, focusing on strengths, questioning thought patterns and beliefs, as well as providing a supportive sounding board and partnership. These strategies increase one’s internal locus of control, promote resilience, and provide ballast against the demands of professional life in an era of diminishing external control.

Although coaching deserves further study, its efficient, results-oriented approach could prove valuable to physicians and health systems worldwide. Hospitals, medical schools, insurers, and practices could consider coaching to improve physician quality of life. Given increasing external pressures, if we hope to lessen physician burnout, we must provide supports that strengthen the internal locus of control and provide a stigma-free method of ameliorating burnout.

## Footnotes

1 Dr. Greenley is a composite profile of typical physician coaching clients.
